# A Study to Assess the Effectiveness of Cryotherapy in Preventing Hematoma at the Arterial Puncture Site Among Post-percutaneous Coronary Intervention (PCI) Patients in a Selected Tertiary Care Hospital, Belagavi

**DOI:** 10.7759/cureus.80921

**Published:** 2025-03-20

**Authors:** Honnagouda Patil, Vishwanath Hesarur, Santoshini Mahanta

**Affiliations:** 1 Department of Nursing, KLE Academy of Higher Education and Research (KAHER) Institute of Nursing, Belagavi, IND; 2 Department of Cardiology, Jawaharlal Nehru Medical College, KLE Academy of Higher Education and Research (KAHER), Belagavi, IND

**Keywords:** bleeding risk, coronary disease, cryotherapy, hematoma, percutaneous coronary intervention

## Abstract

Background: Coronary artery disease (CAD) diagnosis and management have significantly advanced with percutaneous coronary intervention (PCI), yet the procedure remains associated with notable vascular complications in patients, with hematoma being one of the most common post-procedural adverse events. This study aimed to evaluate the effectiveness of cryotherapy in preventing and reducing hematoma formation at arterial puncture sites in patients undergoing PCI.

Methodology: A randomized controlled trial was conducted at a tertiary care center in India, involving 70 patients undergoing PCI via the femoral approach. Patients were randomly divided into experimental and control groups. The experimental group received cryotherapy through ice pack application at two-minute intervals for 10 minutes immediately after sheath removal, while the control group followed the standard hospital protocol. Hematoma was assessed and graded by size, with measurements taken before and after intervention.

Results: The mean age of the patients in the control and experimental groups was 57.29 ± 10.03 years and 57.09 ± 9.67 years, respectively, with a marked male predominance of 57 (81.43%) across both groups. Before the intervention, 18 (51.43%) patients in the experimental group had no hematoma, while 9 (25.71%) patients experienced severe hematoma, and 4 (11.43%) patients had mild hematoma. After receiving cryotherapy, a total of 33 (94.29%) patients in the experimental group had no hematoma and only 2 (5.71%) of patients had mild hematoma. Hematoma reduction was significantly greater in the experimental group (95.35%, *P* = 0.0003) than in the control group (78.79%, *P* = 0.0023). Notably, body mass index (BMI) and antiplatelet drug use showed significant correlations with hematoma formation in the experimental group.

Conclusions: Hence, cryotherapy can be effectively used for the prevention and management of hematoma after sheath removal in post-PCI patients.

## Introduction

Cardiovascular disease (CVD) represents the leading cause of morbidity and mortality among developed countries, which accounts for almost one-third of the total deaths [1]. The two main types of cardiac catheterization techniques used for diagnosing and treating coronary artery disease (CAD) are coronary angiography and percutaneous coronary intervention [2].

The incidences of vascular complications during PCI lead to potential adverse events such as bleeding, hematoma, ecchymosis, pseudoaneurysm, retroperitoneal bleeding, and arteriovenous fistula can significantly impact patient care. These complications not only extend hospital stays but also escalate treatment expenses, underscoring the importance of careful procedural management and post-intervention monitoring [3].

Among all, hematoma is one of the most common vascular complications following PCI, typically arising from incomplete closure of the puncture site and potential tissue damage during sheath removal which causes accumulation of blood and can pose significant clinical challenges, potentially escalating into more serious medical complications if not managed appropriately [4]. Nurses utilize various non-pharmacological interventions, such as sand pillows and cold compresses, to effectively reduce hematoma risks in clinical settings. These techniques provide mechanical support and temperature-based healing, minimizing tissue trauma and promoting optimal patient recovery. Cryotherapy reduces pain and tissue damage by slowing nerve impulses and promoting vasoconstriction. Temperature-induced vasoconstriction reduces blood flow, minimizing bleeding and swelling while promoting healing in injured areas through precise neurological and vascular mechanisms [4]. Hence, the present study investigates the potential of cryotherapy as a targeted intervention to reduce hematoma formation in patients following PCI at the arterial puncture site and rule out the correlation of demographic and clinical variables with hematoma status.

## Materials and methods

Study design and population

This randomized controlled study was conducted at a tertiary care center in India, involving 70 patients who underwent percutaneous coronary intervention (PCI) via the femoral approach in the intensive coronary care unit (ICCU). The patients were randomly divided into experimental and control groups, with 35 (50%) patients in each group. Hemodynamically stable patients and those who could tolerate cryotherapy were included in the study. Patients with coagulopathy were excluded from the study. The study was conducted from August 2022 to July 2023.

Ethical approval

The study was approved by the Institutional Ethics Committee of the institute and was conducted in accordance with the Declaration of Helsinki. Written informed consent was obtained from all participants.

Data collection

In the present study, cryotherapy was given to the patients in the experimental group by applying an ice pack to the puncture site immediately after sheath removal. The ice pack was applied at two-minute intervals for a total duration of 10 minutes. A measuring tape was used to measure the size of the hematoma in centimeters. Hematoma grading was based on its size: a hematoma measuring less than 5 cm was classified as mild, a size between 5 and 10 cm was classified as moderate, and a hematoma larger than 10 cm was classified as severe. In the control group, standard sheath removal was performed according to the hospital protocol. Pressure dressing and sandbag application were performed in both groups, and a post-test was conducted the following day after pressure dressing removal.

Definition

A hematoma represents an accumulation of blood and related cellular components located beneath the skin's subcutaneous tissue. While frequently observed after surgical procedures involving sutures or incisions, hematomas can also develop because of physical trauma. Specifically, these blood collections are likely to form when external forces such as shearing or blunt impact are applied to the skin, or when significant soft tissue damage occurs, including injuries to muscles, tendons, or ligaments [5]. The clinical significance includes potential complications, extended hospital stays, and increased treatment expenses.

Outcomes

This study aims to evaluate the effect of cryotherapy in the prevention of hematoma and its size reduction among post-PCI patients and determine the relationship between pre-test hematoma levels in the control and experimental groups with clinical and demographic variables.

Statistical analysis

The data were analyzed using SPSS software Version 21 (IBM Corp., Armonk, NY). Categorical data were expressed as numbers and percentages and compared using chi-square tests. Continuous variables were expressed as mean and standard deviation (SD) and compared using the Mann-Whitney U test and the Wilcoxon matched-pair test. A *P*-value of <0.05 was considered statistically significant.

## Results

The mean age of the patients in the control and experimental groups was 57.29 ± 10.03 and 57.09 ± 9.67 years, respectively. All the patients underwent PCI through the femoral region.

Table 1 illustrates the demographic characteristics of the control and experimental groups. Gender distribution revealed a marked male predominance, with 57 (81.43%) patients being male across both groups - 30 (85.71%) in the control group and 27 (77.14%) in the experimental group. Age distribution was relatively uniform across the groups, with 19 (27.14%) patients aged ≤50 years, 25 (35.71%) patients aged 51-60 years, and 26 (37.14%) patients aged ≥61 years. Chi-square analysis demonstrated no statistically significant differences in age distribution between the control and experimental groups (χ² = 0.2460, *P* = 0.8840). Body mass index (BMI) analysis revealed that 34 (48.57) patients had normal BMI, whereas,28 (40) patients were overweight and 8 (11.43) patients were obese. While the control group had a higher percentage of overweight patients (18, 51.43%) compared to normal weight (15, 42.86%), the experimental group demonstrated more patients with normal weight (19, 54.29%) and fewer overweight patients (10, 28.57%). In the control group, 2 (5.71) patients were obese, whereas 6 (17.14) were obese in the experimental group. The chi-square test of BMI distribution showed marginal significance (χ² = 4.7560, *P* = 0.0930).

**Table 1:** Demographic characteristics of the control and experimental groups. Data are presented as *n* (%). BMI, body mass index

Variables	Control group (*n* = 35 patients), *n* (%)	Experimental group (*n* = 35 patients), *n* (%)	Total (*N* = 70 patients), *n* (%)	Chi-square test	*P*-value
Male	30 (85.71)	27 (77.14)	57 (81.43)	NA	NA
Age (years)					
≤50	10 (28.57)	9 (25.71)	19 (27.14)	0.2460	0.8840
51-60	13 (37.14)	12 (34.29)	25 (35.71)		
≥ 61	12 (34.29)	14 (40)	26 (37.14)		
BMI (kg/m^2^)					
Normal	15 (42.86)	19 (54.29)	34 (48.57)	4.7560	0.0930
Overweight	18 (51.43)	10 (28.57)	28 (40)		
Obese	2 (5.71)	6 (17.14)	8 (11.43)		

Table 2 demonstrates the assessment of hematoma status before and after the cryotherapy was given to the patients. Before therapy, in total, 40 (57.14) patients had no hematoma, 7 (10) had mild hematoma, 6 (8.57) had moderate hematoma, 11 (15.71) had severe hematoma, and 6 (8.57) suffered from bleeding (*P* = 0.4177, *Z* = 0.8105). In the control group, patients without hematoma increased from 22 (62.86%) initially to 28 (80%) after standard care. Conversely, patients with moderate hematoma changed from 3 (8.57%) before intervention to 7 (20%) following the standard protocol, suggesting minimal improvement with routine management. In the experimental group, the number of patients without hematoma showed significant improvement, increasing from 18 (51.43%) before therapy to 33 (94.29%) after cryotherapy. Before therapy, the experimental group exhibited more severe hematoma conditions than the control group, with 9 (25.71%) patients experiencing severe hematoma, 2 (5.71%) patients having moderate hematoma, 4 (11.43%) patients presenting with mild hematoma, and bleeding occurring in 2 (5.71%) patients. After therapy, hematoma severity in the experimental group significantly decreased, with no patients remaining in the severe or moderate categories. Mild hematoma cases reduced from 4 (11.43%) to 2 (5.71%) patients (*P* = 0.3068, *Z* = 1.0219). The Mann-Whitney U test was used to compare hematoma status between the groups.

**Table 2:** Comparison of hematoma status between the control and experimental groups. Data are presented as *n* (%). The *Z*-value represents the Mann-Whitney U test value.

Hematoma status	Control group (*n* = 35 patients), *n* (%)	Experimental group (*n* = 35 patients), *n* (%)	Total (N = 70 patients), *n* (%)	*Z*-value	*P*-value
Before therapy					
No hematoma	22 (62.86)	18 (51.43)	40 (57.14)	0.8105	0.4177
Mild	3 (8.57)	4 (11.43)	7 (10)		
Moderate	4 (11.43)	2 (5.71)	6 (8.57)		
Severe	2 (5.71)	9 (25.71)	11 (15.71)		
Bleeding	4 (11.43)	2 (5.71)	6 (8.57)		
After therapy					
No hematoma	28 (80)	33 (94.29)	61 (87.14)	1.0219	0.3068
Mild	7 (20)	2 (5.71)	9 (12.86)		

Table 3 describes the analysis of hematoma changes between the groups. Here, the Wilcoxon matched-pair test results showed a statistically significant change in hematoma size before and after therapy in both groups (*P* < 0.05). In the control group, the mean change was 0.74 ± 1.29, representing a percentage change of 78.79%. This change was statistically significant (Z = 3.0447, *P* = 0.0023). In the experimental group, the mean change was 1.17 ± 1.40, representing a percentage change of 95.35%. This change was also statistically significant (Z = 3.6214, *P* = 0.0003).

**Table 3:** Comparative analysis of hematoma changes between control and experimental groups. The Z-value represents the Wilcoxon matched-pairs test value.

Groups	Changes from	Mean ± SD	Percentage of change	Z-value	*P*-value
Control group	Before to after	0.74 ± 1.29	78.79%	3.0447	0.0023
Experimental group	Before to after	1.17 ± 1.40	95.35%	3.6214	0.0003

Table 4 shows the correlation of demographic variables such as age, gender, BMI, comorbidity, and clinical variables, which include anticoagulant drugs and antiplatelet drugs, with hematoma status in the experimental group. BMI (*P* = 0.034) and antiplatelet drugs (*P* = 0.020) showed a significant relationship with the hematoma status in the experimental group. No significant correlation was observed between demographic and clinical variables and hematoma size among patients in the control group.

**Table 4:** Correlation of demographic and clinical variables with the status of hematoma in the experimental group.

Variables	Chi-square test	Degrees of freedom	Significance
Age	9.446	8	0.306
Gender	1.591	4	0.810
BMI	16.690	8	0.034
Comorbidity	25.922	24	0.357
Anticoagulant drugs	7.159	4	0.128
Antiplatelet drugs	18.146	8	0.020

## Discussion

According to our study findings, cryotherapy was a highly effective non-pharmacological treatment in managing hematoma in patients undergoing PCI. Multiple studies have demonstrated that the application of cold therapy serves as an effective method for achieving hemostasis and preventing hematoma formation at arterial puncture sites. The therapeutic mechanism primarily operates through localized vasoconstriction - when cold is applied to the affected arterial site, it triggers a reduction in local blood flow, thereby significantly diminishing the likelihood of hemorrhage and subsequent hematoma development. This physiological response aligns with our findings where cryotherapy demonstrated substantial efficacy in hematoma prevention and management post-PCI [6]. The therapeutic application of cold has a well-established history as an effective non-pharmacological intervention for hemorrhage control. The physiological basis of cryotherapy's effectiveness lies in its ability to induce arterial vasoconstriction, which leads to a cascade of beneficial effects: reduced peripheral blood flow, decreased histamine release and inflammatory response, diminished muscular spasm, and slowed nerve conduction velocity. These mechanisms collectively contribute to the significant reduction in hematoma formation [7]. The therapeutic efficacy of cryotherapy can be attributed to its effect on blood viscosity and flow dynamics at the intervention site. By increasing blood viscosity and subsequently reducing blood flow to the affected area, ice pack application promotes more effective hemostasis. This mechanism appears to play a crucial role in minimizing vascular complications, particularly hematoma formation, as demonstrated by our findings where the experimental group showed significantly better outcomes compared to standard care alone [6].

Kurt and Kaşıkçı investigated the effect of cold application on hematoma, ecchymosis, and pain at the catheter insertion site in patients undergoing PCI and revealed a statistically significant reduction in hematoma formation after applying a cold pack to the femoral artery region following catheter removal and the size of developing hematomas also significantly reduced (*P* < 0.001) [8]. In concordance with this, the current study demonstrated that the majority of patients in the experimental group exhibited no hematoma. Moreover, the number of patients with mild hematoma significantly decreased, with no patients experiencing moderate or severe hematoma after cryotherapy intervention. The hematoma size showed a statistically significant change before and after the intervention. The control group exhibited a percentage change of 78.79% (*P *= 0.0023), while the experimental group showed a more substantial percentage change of 95.35% (*P *= 0.0003) following cryotherapy application. It was concluded that implementing a designed clinical guideline for femoral artery sheath removal significantly reduced complications after cardiac catheterization. The study group showed a significant reduction in hematoma, bleeding, ecchymosis, back pain, and vasovagal reflex (*P* = 0.001) [9].

A study conducted to compare the effectiveness of sand pillows and cold compresses in reducing hematomas in post-PCI patients concluded that cold compresses and cold packs induce vasoconstriction, slow blood flow, and promote clot formation. Hence, they proved to be more effective than sand pillows [10]. Valikhani et al. conducted a randomized clinical trial to evaluate the effect of simultaneous sand-ice bag application on hemorrhage and hematoma following PCI. The study found that the simultaneous application of sand and ice bags to the procedural area significantly reduced the risk of hemorrhage and hematoma in patients post-intervention [11].

A study was conducted to determine the effect of a direct cold compress applied after femoral arterial sheath removal following PCI. The researchers found that the local vascular complications, including bleeding, hematoma, and ecchymosis, were significantly reduced in the experimental group compared to the control group. The cold compress intervention proved to be an effective method for minimizing post-procedural complications in the experimental group [12].

In a randomized controlled trial conducted by Ginajan et al., the effect of early ambulation with a cold pack on the prevention of bleeding and hematoma complications in post-cardiac catheterization patients was found to be significant [2]. A study was conducted to assess the effect of ice bag application on local vascular complications and low back pain after PCI. The results from the first follow-up showed a non-significant trend (*P* > 0.05), but in the second and total follow-ups, the ice-bag group demonstrated statistically significant results (*P* < 0.001) [13].

Bayındır et al. conducted a study to assess the effect of ice bag application on the femoral region to reduce pain in patients undergoing PCI and concluded that applying cold therapy before removing the catheter, reduced the intensity of pain, and also other local vascular complications [14]. Another study conducted by Pamuk and Özkaraman found that applying a cold sand pack for the first 20 minutes after PCI prevented bleeding, reduced the size of ecchymosis, and decreased pain severity compared to a normal sand pack [15].

Limitations

This study has some limitations that should be considered. First, the relatively small sample size may have affected the statistical power and generalizability of our results. Second, being a single-center study, our study may not be representative of a diverse patient population across different healthcare settings. Finally, future multicenter trials with larger sample sizes and longer follow-up periods can help address issues by assessing late-onset hematoma or re-bleeding risks beyond 24 hours.

## Conclusions

This study demonstrates that cryotherapy is a promising non-pharmacological intervention for reducing hematoma formation after PCI. While our results demonstrate the potential benefits of using a two-minute interval protocol for 10 minutes, future research should explore optimization of the treatment protocol by investigating different durations of cryotherapy application, varying time intervals between applications, alternative cold application methods, and the potential benefits of combining cryotherapy with other interventions. Additionally, larger multicenter trials are needed to validate these findings across diverse clinical settings and patient populations and evaluate long-term outcomes. Such studies could help establish standardized protocols for incorporating cryotherapy into post-PCI care guidelines.
